# The Godmother Project: A Virtual Initiative to Support Pregnant and Postpartum Women in Brazil During the COVID-19 Pandemic

**DOI:** 10.9745/GHSP-D-22-00500

**Published:** 2023-04-28

**Authors:** Juliana S. Amaro, Marcia Rochelle T. F. B. Pessalli, Lissandra B. da Cunha, Ana Pilar Betrán, Maria Regina Torloni, Monica M. Siaulys

**Affiliations:** a Communication and Marketing Department, Grupo Santa Joana, São Paulo, Brazil.; b Department of Health Education and Training, Grupo Santa Joana, São Paulo, Brazil.; c UNDP/UNFPA/UNICEF/WHO/World Bank Special Programme of Research, Development and Research Training in Human Reproduction, Department of Sexual and Reproductive Health and Research, World Health Organization, Geneva, Switzerland.; d Research Department, Hospital e Maternidade Santa Joana, São Paulo, Brazil.; e Board of Directors, Grupo Santa Joana, São Paulo, Brazil.

## Abstract

**Background:**

The COVID-19 pandemic increased maternal distress and demand for social support and educational services, while restriction measures decreased the availability of these services.

**Project Description:**

The Godmother Project, a person-centered, WhatsApp-based support and health education initiative, was created after all in-person perinatal educational activities offered at 3 Brazilian hospitals were canceled in 2020. The project was developed in response to pregnant/postpartum women who called the hospitals with questions for health care professionals (HCPs). Health education nurses teamed up with staff from the communication department to design a project to (1) provide a direct communication channel between women and HCPs to answer health-related questions, (2) offer reliable virtual health education material, (3) identify women in need of additional assessment/referrals, (4) offer each woman continuous support from a specific nurse educator (Godmother), and (5) offer a virtual forum where women going through similar experiences could interact.

**Project Development and Rollout:**

The project provides direct communication between WhatsApp groups of 25–30 pregnant/postpartum women and a Godmother. It also offers access to a virtual library of educational materials prepared in response to women’s needs/demands. The project, which began in July 2020 with 1 WhatsApp group of 5 women and 1 Godmother, currently has 305 groups, 6,942 active participants, and 4 Godmothers. Enrollments and WhatsApp groups continued to increase despite reestablishment of in-person educational activities in 2022. The vast majority of 232 participants surveyed in December 2021 were very satisfied with the project, the Godmothers, and the educational materials.

**Conclusions:**

Our findings suggest that WhatsApp groups of pregnant/postpartum women led by dedicated nurse educators can be an important tool to educate and support women during the perinatal period. This type of initiative may be especially important in contexts of physical distancing requirements or situations where social support is unavailable.

## Abstract (precis)

WhatsApp groups of pregnant and postpartum women led by dedicated nurse educators can help educate and support women during the perinatal period, especially in contexts of physical distancing requirements or where social support is unavailable.

## Teaser

**Key Findings:**

- A WhatsApp-based health educational initiative was designed to provide support and health information to Brazilian pregnant and postpartum women after in-person educational activities were canceled during the pandemic.
- The project was very well received by women, and the number of participants continues to grow despite the reinstatement of in-person educational activities.
- The welcoming, supportive attitude of the nurse educators managing the WhatsApp groups is key to ensuring successful interactions with and between the women.
- Due to its success, the project was incorporated into routine antenatal services and is now offered to all women interested in perinatal education and support, along with the in-person educational sessions, as part of a blended approach.

**Key Implications:**

- Program managers may need to train the nurse educators who will lead the WhatsApp groups to ensure that they use appropriate language and a warm, positive attitude when interacting with the women.
- Program managers should consider creating a virtual library where women can easily access high-quality, evidence-based information in lay language.
- In settings where nurse educators are scarce, program managers should consider other trained health professionals for this role.

## BACKGROUND

Becoming a mother is a complex interactive process associated with conflicting feelings and challenges. It requires women to adapt to the physical and hormonal changes taking place in their bodies, go through new experiences, acquire knowledge, and develop novel social roles throughout pregnancy, delivery, and the postpartum period.^1^^,^^2^ Expectations and uncertainties related to pregnancy, birth, and the care of a new baby can lead women, especially nulliparas, to become anxious and stressed.^3^ Feeling safe and supported can increase women’s resilience to the natural stressors of pregnancy.^3^ Ideally, all pregnant women should have easy access to a trusted health professional to listen to their worries and answer their questions. However, antenatal care (ANC) visits are often brief and far apart, and health care providers (HCPs) may not have the time or training to fully meet these demands. Moreover, in many settings, group educational activities are not routinely offered or available during ANC. This leads many women to seek information and support elsewhere, such as magazines, the Internet, social media, and virtual women’s groups.^4^^,^^5^ However, the quality and comprehensiveness of the information available in these channels is heterogeneous and often limited, inaccurate, unreliable, and unverified.^6^^–^^12^ This situation can lead some women to become frustrated and more anxious or to have an incomplete or biased view of pregnancy, childbirth, and the postpartum period.

The COVID-19 pandemic, the restrictions and social isolation measures that were imposed, and the need to reconfigure maternity care and reallocate HCPs affected the access to and quality of obstetric services worldwide.^13^^–^^16^ This situation, along with the fear of maternal/neonatal contamination^17^ and the information overload about the pandemic (“infodemic”),^18^ increased distress, anxiety, and depression symptoms in pregnant and postpartum women in many countries, including Brazil.^19^^–^^26^ The fears and uncertainties of being pregnant during the pandemic of a new virus, along with the physical isolation imposed by COVID-19, created a unique situation of increased maternal distress and demand for social support and educational services, alongside decreased availability of such services. This challenge led some health care institutions to rapidly implement or scale up virtual perinatal education services. Previous studies reported the feasibility, acceptability, and effectiveness of using social media and mobile health interventions developed by health professionals to inform, support, and educate pregnant and postpartum women in general.^27^^–^^31^ There are fewer publications on the use of these interventions during the COVID-19 pandemic, and the largest studies were conducted in high-income countries.^32^^–^^34^

We describe the development, implementation, and growth of the Godmother Project, a virtual initiative to educate and support Brazilian pregnant and postpartum women during the COVID-19 pandemic. We also report participants’ feedback and lessons learned.

We describe the development, implementation, and growth of a virtual initiative to educate and support Brazilian pregnant and postpartum women during the COVID-19 pandemic.

## GODMOTHER PROJECT DESCRIPTION

### Project Motivation and Objectives

Before the COVID-19 pandemic, all perinatal education courses were offered exclusively in person at ANC clinics in group classes (15–20 women/couples) led by nurse educators. In April 2020, following recommendations from Brazilian public health authorities to reduce the spread of COVID-19, Grupo Santa Joana (GSJ) clinics interrupted all in-person educational activities routinely offered to pregnant and postpartum women. Days after these cancellations, pregnant women began to call the hospitals to talk with an HCP who could answer their questions and concerns related to COVID-19, pregnancy, childbirth, and the postpartum period. The phone operators transferred these calls to the Hospital e Maternidade Santa Joana’s (HMSJ) department of continuous education, where nurse educators answered questions and provided guidance to the women over the phone. As the number of calls increased and São Paulo city went into a complete lockdown, the head of HMSJ’s communication department and nurses from the continuous education department decided to create a specific project to offer person-centered online support and health education to women in the perinatal period (pregnancy and postpartum) because in-person educational activities were cancelled. This initiative was called the “Godmother Project” (*Projeto Madrinha*, in Portuguese), referring to the new role that the nurses responsible for communicating with the women would play in educating, supporting, and comforting the women who voluntarily enrolled in the project.

The Godmother Project did not aim to replace ANC, which continued to be offered online and in person by hospital clinicians throughout the pandemic. Instead, it was designed to achieve the following 5 objectives.

1. Provide a direct communication and contact channel with the institution so that women could have easy and immediate access to an HCP when they wanted information or had questions about pregnancy, childbirth, the postpartum period, or any health-related matters, including COVID-19.
2. Provide women with easily accessible, high-quality, reliable, and trustworthy information and educational materials about pregnancy, childbirth, the postpartum period, and the pandemic.
3. Identify women who, during their interactions with the Godmother, may need a referral to hospital clinicians or specialists (e.g., obstetricians, mental health professionals, or lactation specialists) for additional assessment/guidance.
4. Offer women a warm, supportive, and comforting virtual interaction with their Godmother to foster feelings of calm, safety, and self-competence.
5. Offer a virtual forum where women could interact with and get support from other women who were going through the same experience.

### Setting

The project was created, implemented, and reported on by investigators and staff from HMSJ, one of the largest private maternity hospitals in São Paulo, Brazil, with 190 adult and 100 neonatal beds and an average of 10,000 deliveries per year. HMSJ is a tertiary referral facility for high-risk pregnancies and neonatal surgeries. The hospital is part of the GSJ, which also includes 2 other private maternity hospitals located in São Paulo and 4 outpatient ANC clinics staffed by a multidisciplinary team of 40 HCPs (obstetricians, nurse-midwives, physiotherapists, nutritionists, and psychologists) who manage approximately 4,000 women each year.

Health care costs of most women managed in these ANC clinics and who give birth under the care of obstetricians and nurse-midwives on duty at the 3 hospitals are covered by private health plans paid by women’s employers.

### Participants

The Godmother Project is free of charge and open to all women receiving ANC at any of the 4 GSJ prenatal clinics and to women who have their own private obstetricians but intend to give birth in any of the GSJ hospitals. The project is open to healthy women and to those with any preexisting health condition (e.g., hypertension or diabetes). Initially, pregnant or postpartum women learned about the project when they called the hospital asking to speak with an HCP. As the initiative grew, posters and folders about the project were created and placed in the waiting areas of all ANC clinics.

The project is also presented to pregnant couples/families during routine hospital tours. In the Brazilian private health sector, most pregnant couples/families visit several maternity hospitals before deciding where they will give birth. These in-person or online group visits occur daily in each of the 3 GSJ hospitals and are guided by a hospital clerk.

### Communication Channel

**FIGURE 1:**
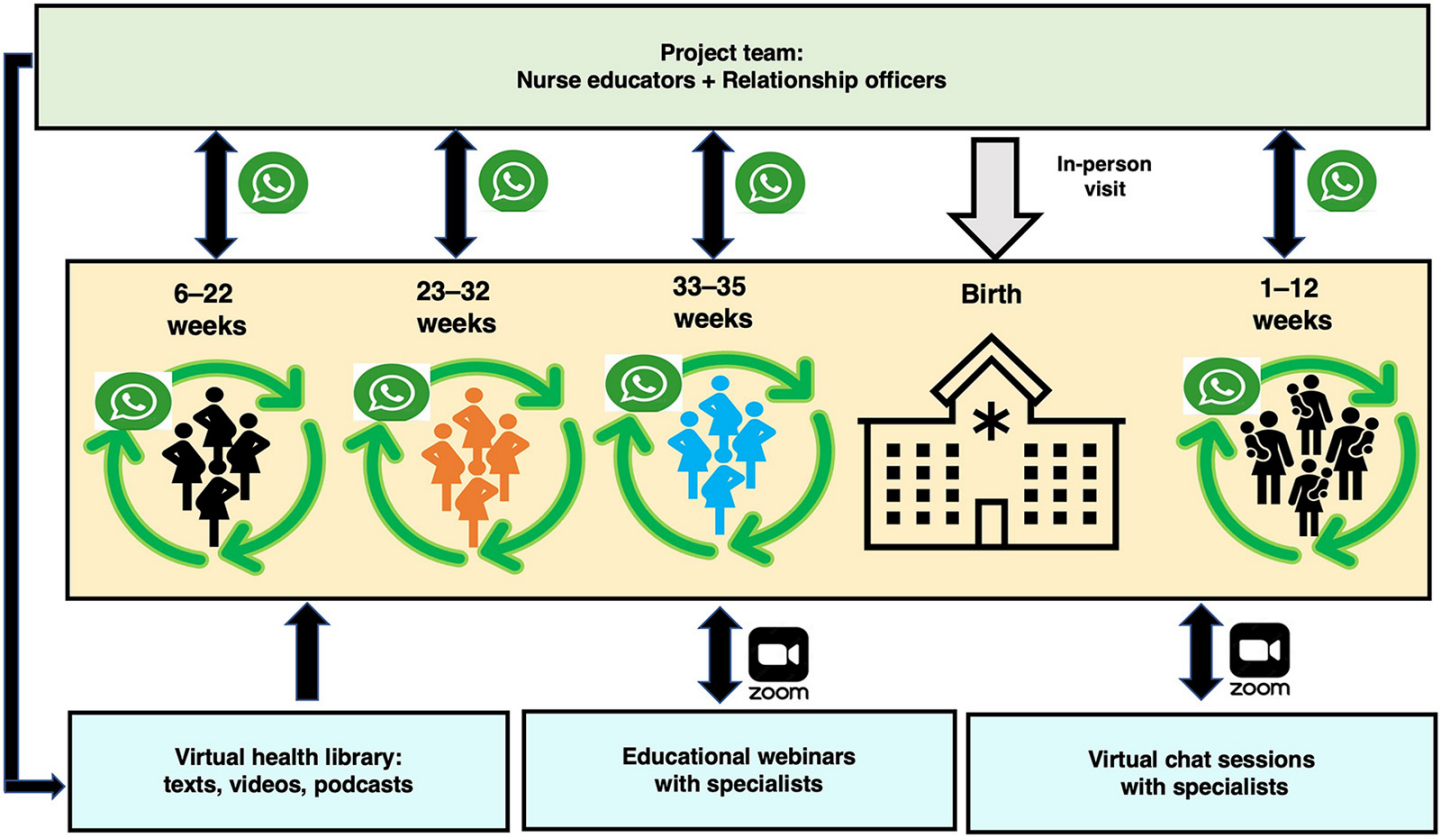
The Godmother Project Ecosystem^a^ ^a^ Green box: staff members; yellow box: participants; blue boxes: material and activities.

The main channel of communication for the project is WhatsApp Messenger (hereafter referred to as WhatsApp) groups. WhatsApp was chosen as the project’s social media platform because it is the free app most downloaded in the country (installed in 99% of Brazilian smartphones^35^) and allows the creation of groups and online social interactions. Upon enrollment, all participants are asked about their current gestational age. Based on this information, women are included in a WhatsApp group with other women of the same approximate gestational age (6–22 weeks, 23–32 weeks, or 33–35 weeks) so that each WhatsApp group has 5–30 women. As more women asked to be included in the project, new groups for each of the 3 gestational ages were created to ensure a maximum of 30 participants per group. The WhatsApp groups end once all participants have given birth and their babies are aged 3 months (Figure 1).

### Staff and Activities

The project team consists of 2 types of professionals: relationship officers and patient education nurses (the Godmothers). All project staff members are trained to use sympathetic and welcoming language in text and phone contacts with the women and to avoid excessive use of emojis and images in WhatsApp interactions. Conversations between project staff members and the women are personal and informal but respectful. Project staff members are immediately informed by the hospital administration whenever a project participant presents at the hospital’s emergency department or is admitted, for any reason, in any of the 3 hospitals.

All project staff members are trained to use sympathetic and welcoming language in text and phone contacts with the women.

#### Relationship Officers

Relationship officers are administrative clerks originally employed by the hospital for other activities and who had part of their working time allocated to the project. Over time, some relationship officers started to devote all their working hours to the project. All relationship officers receive training on how to create, run (add/exclude participants), and dissolve WhatsApp groups; collect relevant project statistics; and interact with participants about administrative issues.

Relationship officers make the first contact with each new potential participant through a personal phone call, welcome the woman, explain how the project works, ask her to complete the registration form, and assign her to a gestational age-appropriate WhatsApp group. Before enrollment, each woman receives a text message about the confidentiality of personal information provided on her registration form, including that it will not be shared with anyone outside of the Godmother Project staff team and will be deleted at the end of her participation. The text also informs her that she has the option of using a nickname in the WhatsApp group and that all questions or opinions that she posts in the group will only be used for educational purposes within the project. Enrollment only occurs after the woman indicates that she has read, understood, and agreed with these conditions.

Relationship officers are responsible for organizing waiting lists, creating and administrating new WhatsApp groups up to their dissolution, monitoring project statistics, and answering women’s questions about administrative issues (e.g., list of documents needed for hospital admission, how to schedule a consultation with a hospital clinician, and visiting hours). Relationship officers also interact personally with the women when they are in the emergency department or are admitted to the hospital to give birth (Figure 1). The objective of these in-person contacts is to welcome and support the woman and facilitate her interaction with other hospital staff, up to discharge. This service is offered only for women enrolled in the Godmother Project.

Godmothers are senior obstetric nurses originally employed by the hospital to work in the continuous education and training department.

#### Godmothers

Godmothers are senior obstetric nurses originally employed by the hospital to work in the continuous education and training department who had part of their working time allocated to the project. Over time, due to the increase in the number of project participants, these nurses stopped working on other activities and began working exclusively on the project. Their salaries and working hours did not change. Those who work extra time managing WhatsApp interactions or other project activities receive additional payment for these hours. The selection of Godmothers involves a process that assesses specific characteristics and skills, including empathy, communication, interpersonal relation, reflection and interpretation capacity, and emotional maturity. Those who are approved undergo a standard 6-hour training module and spend 1 week being mentored by a senior Godmother. All Godmothers have daily contact with the project manager and attend a weekly team meeting for updates and to share experiences.

Each Godmother calls every new project participant allocated to her WhatsApp group to welcome the woman, introduce herself, and explain how she can help and how and when she can be reached privately (via phone or text message) in case the woman has questions or concerns that she does not want to post on the WhatsApp group. The Godmother also reinforces the importance and value of women’s opinions, views, and questions posted in the WhatsApp groups and the safety and privacy of these groups. Godmothers moderate their WhatsApp groups continuously throughout the day to address misinformation posted by the women and answer (in text messages) all health-related questions posted by participants. To complement answers to women’s questions, the Godmothers post links to virtual educational materials created specifically for the project and inform participants about upcoming live educational webinars and virtual chat sessions (question-and-answer meetings) with hospital specialists (e.g., anesthetists, neonatologists, and nutritionists) scheduled exclusively for project participants (Figure 1).

Godmothers moderate their WhatsApp group, answer questions, and share virtual educational materials with women.

The Godmothers work with a team of hospital clinicians and communication specialists to create, update, and curate an online library of educational health materials for laypersons in various formats (texts, infographics, videos, and podcasts) based on the best available scientific evidence. The main sources of information are guidelines from the World Health Organization, Brazilian Society of Obstetrics and Gynecology, Brazilian Society of Pediatrics, and Brazilian Ministry of Health. The Godmothers are also trained to identify women who may need additional attention/referral to specialists because of the messages posted in their WhatsApp group. When she suspects that a woman in 1 of her WhatsApp groups could need additional attention, the Godmother makes a personal phone call to that woman, explains her concerns, and offers additional support/referrals for specific perceived needs. Finally, after project participants in their WhatsApp groups give birth, Godmothers visit them in person before they are discharged from the hospital (Figure 1). During these visits, Godmothers answer women’s questions and provide support related to maternal/neonatal needs.

### Ethical Approval

The project was approved by the HMSJ institutional review board (57419622.8.0000.5443). Informed consent of survey respondents was waived due to its anonymous nature.

## PROJECT DEVELOPMENT AND ROLLOUT

### Acceptance of the Project

The first WhatsApp group was launched in July 2020 with 5 pregnant women, 1 Godmother, and 1 relationship officer. At first, the project only offered women access to a Godmother (via WhatsApp) to answer their questions. A few months later, participants were given access to virtual educational material (texts, infographics, videos, and podcasts) created specifically for them. Later, the project added presential contacts with the participants when they were admitted to 1 of the hospitals, including postpartum visits. Finally, the project created live webinars and chat sessions. Due to the growing number of interested women, new groups were created over the following months, and additional nurse educators and officers were recruited to help run these groups. Currently, 7 professionals (4 nurses and 3 administrative clerks) employed by the GSJ dedicate all of their working hours (8 am to 6 pm, 7 days/week) to run the Godmother Project and offer daily online education and support to pregnant and postpartum women enrolled in the more than 300 WhatsApp groups.

**FIGURE 2:**
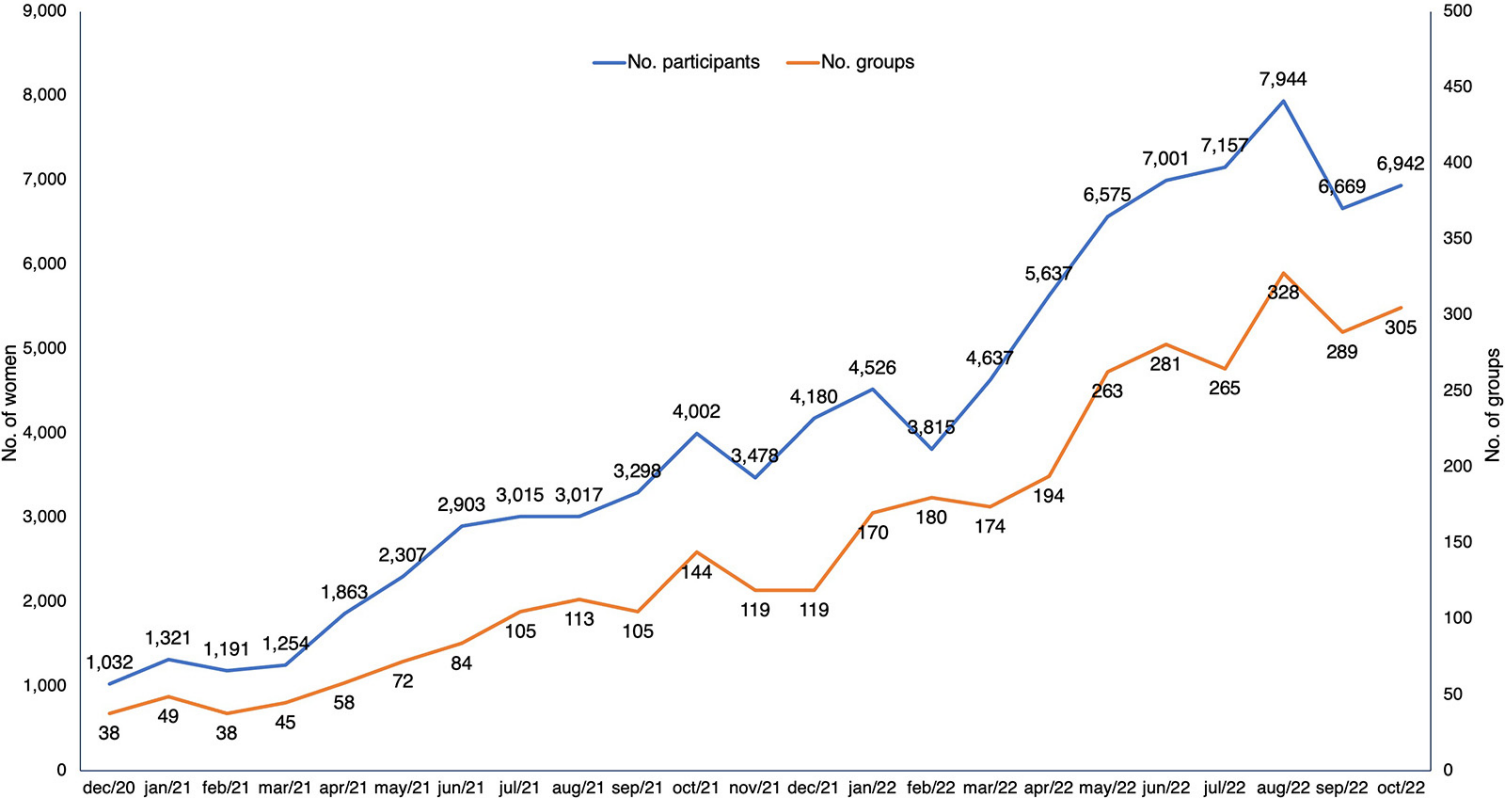
Monthly Number of Groups and Pregnant or Postpartum Women Participating in the Godmother Project

We began collecting official project statistics in December 2020. With the reduction in the number of severe COVID-19 cases as a result of mass vaccination and the gradual lifting of restrictions for in-person group activities, in April 2022, the 4 GSJ clinics resumed all routine in-person educational activities, including antenatal, postpartum, and lactation courses. We expected that the return of these in-person activities would lead to a gradual decrease of new enrollments in the Godmother Project. Surprisingly, this did not occur, and the number of interested women and new groups continued to increase (Figure 2).

As of October 2022, there were 305 ongoing groups with 6,942 active participants (Figure 2); up to that date, the project had included a total of 20,175 unique participants (pregnant women). The project’s virtual health library started with 30 short texts/infographics and currently comprises approximately 850 educational materials (500 texts/infographics, 250 videos, and 80 podcasts).

### Changes and Adaptation to Fulfill Emerging Needs

In November 2021, in response to women’s suggestions, the project began hosting live interactive webinars (via Zoom) with HCPs (obstetricians, anesthesiologists, neonatologists, obstetric nurses, and lactation specialists) who work in 1 of the 3 GSJ hospitals. During these webinars, an HCP gives a 30-minute presentation about a predefined specific topic within their area of expertise (e.g., a neonatologist will give a presentation about “neonatal jaundice”), which is followed by a 30-minute period to answer women’s written or oral questions on the topic. The project currently holds weekly live educational webinars open to all women enrolled in the project (Figure 1). In December 2021, in response to women’s demand, the project also started to hold prescheduled interactive chat sessions via Zoom, where 1 HCP is available to answer questions on any topic within their area of expertise (e.g., if the invited HCP is a neonatologist, they will answer any question related to babies’ health). These virtual chat sessions last 60–90 minutes and are dedicated exclusively to answering questions from the women about any topic related to the HCP’s specialty (Figure 1).

In response to women’s suggestions, the project began hosting educational webinars and interactive online chat sessions.

### Women’s Reaction to the Project

**FIGURE 3:**
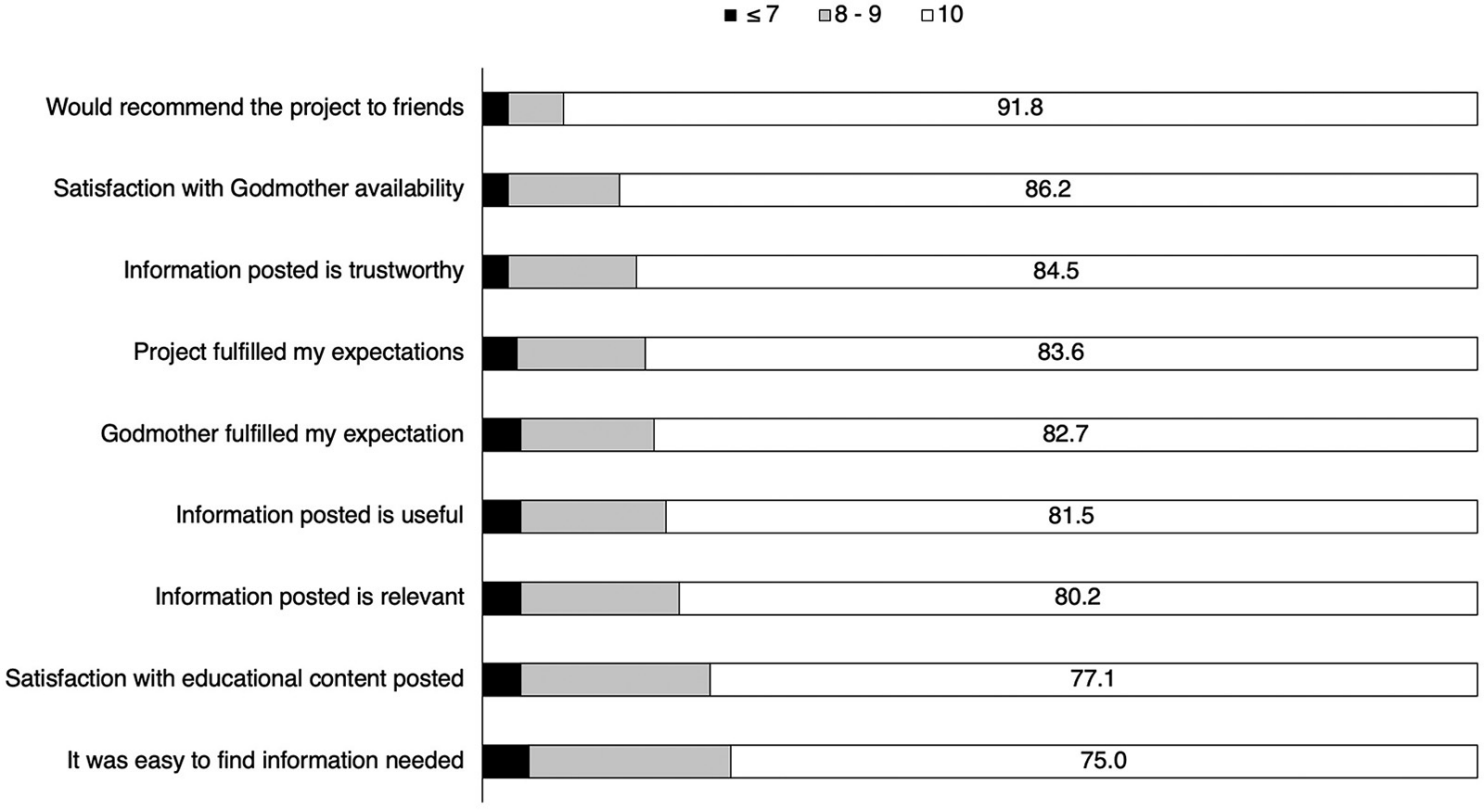
Satisfaction^a^ of Participants With the Godmother Project (N=232) ^a^ How much respondent agreed with each statement (0: total disagreement, 10: total agreement). Numbers represent the percentage of participants who selected each score.

In December 2021, we formally conducted an anonymous web-based survey (during 1 week) to assess women’s reactions to the Godmother Project. An invitation to answer the survey was posted in all WhatsApp groups. Women who voluntarily agreed to participate clicked on a link that led to an online questionnaire (via SurveyMonkey) with open and closed questions. The first section collected information on participants’ sociodemographic characteristics, obstetric history, and enrollment in the project. The second section asked participants to (1) rate the educational material available and their overall satisfaction with the project and the staff who ran it (using Likert scales), (2) describe the most and least appreciated features of the project (free-text answers), and (3) write general comments about their experience in the project and suggestions for improvement (free-text answers). We categorized free-text answers into major themes. The questionnaire was created by the authors, tested in a pilot group of volunteers (women who were no longer active but had participated in WhatsApp groups), and modified to ensure clarity. Survey participants did not receive any incentives. Table 1 presents the main characteristics of the 232 respondents. Respondents were aged 21–46 years and were mostly white, married, employed, and had more than 12 years of education. Most respondents were also pregnant (7–40 weeks) and multiparous. The most popular pregnancy, labor and delivery, postpartum, and neonatal topics were fetal development, preparing for labor and birth, breastfeeding, and caring for the newborn, respectively. The preferred formats for presenting health topics were short texts, short videos, and images/infographics (Table 2). Nearly 92% would certainly recommend the project to friends, and the overwhelming majority were satisfied with the availability of Godmothers and agreed that the information posted by Godmothers was trustworthy, useful, relevant, and easy to retrieve (Figure 3). According to the answers provided in free text, the most appreciated feature of the project was the welcome, support, attention, and availability of the Godmothers (Table 3). Comments made by the respondents were mostly positive (Box).

**TABLE 1.** Characteristics of Survey Respondents Who Participated in the Godmother Project, São Paulo, Brazil

**Characteristic**	**No. (%)** **(N=232)**
Age, years	
<25	13 (5.6)
25–34	93 (40.1)
35 or more	126 (54.3)
Race (self-reported)	
White	143 (61.7)
Mixed^a^	65 (28.0)
Asian	13 (5.6)
Black	11 (4.7)
Marital status	
Married	185 (79.8)
Cohabitating	35 (15.1)
Other	12 (5.1)
Education	
<12 years	4 (1.7)
12 years	21 (9.1)
>12 years	207 (89.2)
Current employment	
Employed	160 (69.0)
Unemployed	33 (14.2)
On maternity leave	39 (16.8)
Current reproductive status	
Pregnant	168 (72.4)
Postpartum	64 (27.6)
Gestational age, weeks (n=168)	
<12	3 (1.8)
12–28	47 (28.0)
>28	118 (70.2)
Postpartum period, months (n=64)	
1	38 (59.4)
2–3	26 (40.6)
Parity	
0	82 (35.3)
1 or more	150 (64.7)
Previous cesarean delivery (n=150)	
0	66 (44.0)
1	71 (47.3)
2 or more	13 (8.7)

a Black and White.

**TABLE 2.** Most Popular Topics Covered in the Godmother Project Among Survey Participants, São Paulo, Brazil

**Topic**	**No. (%)**^a^ **(N=232)**
Pregnancy	
Development of the fetus	85 (36.6)
Care of breasts in pregnancy	73 (31.5)
Mental health in pregnancy	46 (19.8)
Antenatal care tests	41 (17.7)
Vaccines in pregnancy	36 (15.5)
Fathers’ role	27 (11.6)
Labor and delivery	
Preparing for labor and vaginal birth	62 (26.7)
Uterine contractions	58 (25.0)
Analgesia and anesthesia for birth	32 (13.8)
Cesarean birth	28 (12.1)
Hospital routines	22 (9.5)
Postpartum	
Breastfeeding	77 (33.2)
Mental health after delivery	22 (9.5)
Milk collection and storage	16 (6.9)
Going back to work	3 (1.3)
Neonatal/infant	
Caring for the newborn baby	95 (40.9)
Baby’s clothes (layette)	63 (27.2)
Vaccines for the baby	44 (19.0)
Your babýs sleep	43 (18.5)
Preferred formats	
Short written material	197 (84.9)
Short videos (up to 5 minutes)	148 (63.8)
Images/infographics	128 (55.2)
Podcasts	68 (29.3)
Live educational webinars	50 (21.6)

a More than 1 answer is possible.

**TABLE 3.** Most and Least Appreciated Features of the Godmother Project Among Survey Participants, São Paulo, Brazil

	**No. (%)**
Most appreciated aspects of the project (N=141 respondents)^a^	
Welcome, support, attention, and availability of the Godmothers	74 (52.5)
Information content	24 (17.0)
I can ask questions and get answers at any time	20 (14.2)
Interaction and sharing experiences with other women	19 (13.5)
Speed of Godmothers’ responses to women’s questions	4 (2.8)
Least appreciated aspects (N=32 respondents)^a^	
Inconvenient day/time of the live educational webinars	8 (25.0)
Some topics are missing	8 (25.0)
Excessive parallel conversions/messages between participants	7 (21.9)
Lack of interaction between participants	4 (12.5)
I want to interact more privately with my Godmother	3 (9.3)
Other^b^	2 (6.3)

a Free-text answers grouped according to major themes.

b One participant each: “Informative texts are too long”, “My Godmother was substituted.”

BOX Participant Feedback on Godmother Project

“Very useful project. I feel welcome! I really liked the meetings about mental health (during pregnancy and after birth), and breastfeeding.”

“I felt very welcomed. I know that the Godmother Project can give reliable answers to my questions about pregnancy.”

“I liked that I could ask all the questions that I wanted! I also liked the interaction with the Godmothers and the other women (in the WhatsApp group) to share information and our experience.”

“What I most liked was the warm welcome from my Godmother. She was always available to answer my questions. This is very helpful because it’s my first pregnancy.”

“I really like that the Godmother can give personal attention when you need it. I also liked their care and affection, and the interaction with other women in the (WhatsApp) group.

“I can’t participate in any of the live webinars and chat sessions because I work. These sessions should be conducted at times that allow working people to attend (for example, at 7 PM).”

“Godmothers should give us a list of books, magazines, sites, and media channels that have good information about pregnancy and childbirth.”

“I didn’t like that my Godmother didn’t answer my question about (the risk of) taking a medication (in pregnancy). She should give us more direct answers instead of saying ‘Talk with your doctor.’ This happened several times and it bothered me.”

## SUCCESSES, LESSONS LEARNED, AND CHALLENGES

The rapid uptake of this WhatsApp-based initiative by pregnant Brazilian women was a positive surprise and suggests that it fulfilled the need for information and social support and connection heightened by the COVID-19 pandemic. While the rapid acceptance of the WhatsApp groups was a welcome response, the growing demand for new groups was a challenge that required agile organization of the institution to allocate more nurses and clerks to the project team.

Another positive effect of the project has been the strong and lasting bonds established between the Godmothers and most women. This was made evident by the many spontaneous compliments about the Godmothers posted by women in all WhatsApp groups, especially after they gave birth or when they were leaving the group at the end of the postpartum period. Another unexpected effect of the project has been its motivation for other hospital staff to collaborate with the initiative. The project has a waiting list of education nurses and clerks who will be trained and join the team when vacancies arise.

The rapid uptake of the project suggests that it fulfilled the need for information and social support and connection heightened by the COVID-19 pandemic.

One of the first challenges faced by the team was the timely creation and continued update of the virtual health library, which was meant to offer participants high-quality, evidence-based information in lay language on a large range of topics. This activity demanded that the Godmothers dedicate more hours of their time and required the collaboration of other HCPs who worked in the GSJ hospitals and ANC clinics to help create or find, translate, adapt, and validate these materials before they were uploaded to the virtual library. Another challenge was the need to create a specific training program for new staff (nurse educators and relationship officers) who joined the project team. This training had to take into account differences in professionals’ skills, knowledge, experience, and communication styles, while ensuring that all project staff members would interact with WhatsApp participants in the same way (i.e., nonjudgmentally and with competence, confidence, and warmth).

This initiative aligns with the World Health Organization’s recommendations for digital targeted client communication for health issues regarding sexual, reproductive, maternal, newborn, and child health.^37^ While our participants gave good ratings to the educational component of the initiative, the most appreciated feature of the project was the warm attitude and availability of the Godmothers. This finding shows how much pregnant/postpartum women value the “support” component of the initiative and could explain why enrollment in the project did not decrease after the return of regular in-person perinatal educational activities.

In 2016, the World Health Organization highlighted the importance of providing effective social, emotional, and psychological support to pregnant women in a respectful way, alongside effective health education communication, as key ANC functions to improving women’s lives and quality of care.^38^ Social support from family has been linked to maternal psychosocial health,^39^ and online support from other persons, including HCPs, can help increase maternal resilience and self-efficacy and alleviate the stress and anxiety frequently associated with the perinatal period.^40^ Psychological distress during pregnancy is a serious problem that has been associated with adverse maternal and perinatal outcomes, poor maternal psychosocial functioning, breastfeeding and parental difficulties, and poorer socioemotional and cognitive development of the offspring.^41^^–^^46^Although traditional in-person perinatal education courses offer the opportunity to interact with the trainer and other women, the main focus of these classes is to provide practical knowledge about healthy habits during pregnancy, childbirth preparation, and information about breastfeeding and care of the newborn.^47^ Contrary to the person-centered format of the Godmother Project, conventional maternal education classes are usually not designed to attend the specific needs of the participants but rather deliver information and messages that the educators believe are important.^48^ A hybrid model combining in-person perinatal education classes with online person-centered information and support groups led by trained health providers could improve the quality of care offered to pregnant and postpartum women.

A hybrid model of in-person education classes and online person-centered information and support groups led by trained health providers could improve care for pregnant and postpartum women.

In summary, key ingredients for the success of this project were: (1) the use of digital targeted client communication, (2) flexibility to incorporate participants’ suggestions and the constant attention paid to their comments and criticisms, and (3) the recruitment of empathetic Godmothers capable of providing effective emotional and social support as well as health education to pregnant and postpartum women. We think that with these key ingredients available this type of initiative could be successfully replicated in other settings, after adjustments due to differences in culture and communication.

## DISCUSSION

### Similar Initiatives

Pregnant women in other countries also indicated their willingness to use mobile technology to fill their need for information and support during the COVID-19 pandemic. A survey conducted in Turkey during the pandemic on the use of mobile apps by pregnant women reported that 96% of the 376 respondents regarded the use of these apps as beneficial, 73% wanted to get counseling via video conferencing, 91% wanted to exchange ideas with other pregnant women through these apps, and almost all would like to ask questions to a physician/nurse using this technology.^36^ However, to our knowledge, there are no other virtual support and educational initiatives targeted at women in the perinatal period of this nature and magnitude conducted in middle-income countries during the COVID-19 pandemic. Pasadino et al. reported on the successful rapid transition of all in-person antenatal classes offered at New York University hospitals to an online format during the COVID-19 pandemic using mainly prerecorded online webinars or classes that clients could assess at their convenience using Webex. Authors also described the use of Zoom for live interactive webinars and question-and-answer sessions with educators. These live sessions were attended by nearly 90% of the 2,616 participants enrolled in the educational program, and authors underscore the importance of connection to a live educator to address the emotional needs and alleviate the anxiety of pregnant couples during the pandemic. Similar to the Godmother Project, the initiative described by Pasadino et al. was developed and led by nurse educators; however, the educational program charged fees from the women, used a Facebook page to post evidence-based information, and incentivized participants to send questions and concerns to a specific email account.^32^ A small trial involving 44 first-time mothers in Turkey during the pandemic reported that participation in 8 online interactive educational sessions using Microsoft Teams significantly decreased women’s worries about labor, fear of childbirth and fear of COVID-19, and improved their preparedness for labor.^33^ A mixed-method study conducted in a southern Brazilian city involving 30 mothers of toddlers reported that a moderated WhatsApp support group was feasible and acceptable and improved maternal psychosocial well-being during the pandemic.^34^

### Strengths and Limitations

A strong point of our case study was the assessment of users’ reactions by a pretested self-administered questionnaire with simple quantifiable scales and free text boxes to enter written comments, as suggested by the Kirkpatrick approach.^49^ The anonymous nature of the survey improved its objectivity and reduced the probability of courtesy bias.^50^ We acknowledge several limitations of the initiative, starting with the lack of involvement of potential project users (pregnant and postpartum women) in the creation team. However, the feedback, views, opinions, and spontaneous suggestions of active project participants are highly valued and carefully considered when making changes or adjustments to the project. We also acknowledge that the number of survey respondents was small and the possibility that self-selection bias may have influenced results. We admit that the quantitative nature of the survey may have missed some relevant aspects of users’ reaction to the project. Ideally, a mixed-methods study, with a qualitative component (in-depth virtual interviews or focus groups with some women) would allow a better understanding of how women perceive the project. Another limitation was that we did not conduct economic analyses to assess the costs involved in the creation, implementation, and maintenance of the project. Despite the lack of detailed data, we estimate that the main costs for the implementation of the project involved staff selection and training and the need to hire new employees (nurses and clerks) to replace those who became fully dedicated to the project. Finally, we only assessed women’s reaction to the project (Kirkpatrick level 1).^49^ We plan to assess the effects of the project on participants’ knowledge acquisition and attitudes (Kirkpatrick level 2) as well as changes in health behaviors (Kirkpatrick level 3), such as diet and physical activity, after enrollment in the project compared to baseline measures. We also plan to assess differences in clinical outcomes between women who participate in the project versus those who receive standard care (Kirkpatrick level 4).

### Future Directions

The success of the initiative and sustained demand for more groups led the GSJ board of directors, in May 2022, to integrate the Godmother Project as part of the routine services offered to all women managed in its 4 ANC clinics or who intend to deliver in any of the 3 maternity hospitals. In May 2022, in response to suggestions from participants, the project created smaller WhatsApp groups for women with specific characteristics/needs (surrogate pregnancies and women undergoing assisted reproduction). Additional specific groups may be created in the future in response to new demands.

Digital health education and communication tools have long been available, but the COVID-19 crisis provided the impetus to rapidly develop, customize, and expand a digital solution focused on the needs of Brazilian women in the perinatal period. We encourage others to replicate, adapt, and test this virtual perinatal educational and support initiative in different populations. In settings where nurse educators are scarce, investigators could test the effectiveness of involving other trained professionals (e.g., midwives, physician assistants, health officers, or even student nurses) in this role. In settings that cannot pay personnel to manage these groups, carefully selected volunteers could be recruited, trained, and motivated to work by receiving nonfinancial incentives (e.g., educational credits, certificates, or public recognition).

## CONCLUSIONS

Our findings suggest that small WhatsApp groups of pregnant women led by dedicated, trained nurse educators can be an important tool to educate and support women during the perinatal period. The warm, supportive, engaged, and reassuring attitude of the nurse educators who manage the groups is a key component to ensuring successful connections and interactions with and between participants of the WhatsApp groups. This type of initiative is important in contexts of physical distancing requirements or situations where social support from other persons is not available and fills a need for virtual engagement common among modern women even when and where in-person engagement options are available.

## Notes

**Peer Reviewed**

**First published online:** April 17, 2023.
